# Look at the Beat, Feel the Meter: Top–Down Effects of Meter Induction on Auditory and Visual Modalities

**DOI:** 10.3389/fnhum.2016.00108

**Published:** 2016-03-23

**Authors:** Alexandre Celma-Miralles, Robert F. de Menezes, Juan M. Toro

**Affiliations:** ^1^Information and Communication Technologies Engineering (ETIC), Language and Comparative Cognition Group – Center for Brain and Cognition, Universitat Pompeu FabraBarcelona, Spain; ^2^Institució Catalana de Recerca i Estudis AvançatsBarcelona, Spain

**Keywords:** beat perception, meter induction, cross-modal timing mechanisms, music evolution

## Abstract

Recent research has demonstrated top–down effects on meter induction in the auditory modality. However, little is known about these effects in the visual domain, especially without the involvement of motor acts such as tapping. In the present study, we aim to assess whether the projection of meter on auditory beats is also present in the visual domain. We asked 16 musicians to internally project binary (i.e., a strong-weak pattern) and ternary (i.e., a strong-weak-weak pattern) meter onto separate, but analog, visual and auditory isochronous stimuli. Participants were presented with sequences of tones or blinking circular shapes (i.e., flashes) at 2.4 Hz while their electrophysiological responses were recorded. A frequency analysis of the elicited steady-state evoked potentials allowed us to compare the frequencies of the beat (2.4 Hz), its first harmonic (4.8 Hz), the binary subharmonic (1.2 Hz), and the ternary subharmonic (0.8 Hz) within and across modalities. Taking the amplitude spectra into account, we observed an enhancement of the amplitude at 0.8 Hz in the ternary condition for both modalities, suggesting meter induction across modalities. There was an interaction between modality and voltage at 2.4 and 4.8 Hz. Looking at the power spectra, we also observed significant differences from zero in the auditory, but not in the visual, binary condition at 1.2 Hz. These findings suggest that meter processing is modulated by top–down mechanisms that interact with our perception of rhythmic events and that such modulation can also be found in the visual domain. The reported cross-modal effects of meter may shed light on the origins of our timing mechanisms, partially developed in primates and allowing humans to synchronize across modalities accurately.

## Introduction

Metrical structure is fundamental for our perception of rhythm in music. It allows humans to process the temporal events of music in an organized manner. Metrical structure is based on two distinct processes: beat extraction and meter induction (Fitch, 2013). The former consists of extracting an isochronous beat from a stream of events. This results in beats appearing as periodic points over time. The latter consists of the hierarchical organization of these periodic beats into sequences of strong and weak patterns. The downbeat (the perceptually prominent beat) usually occurs at a subharmonic frequency of the beat, such as 2:1, 3:1, or other more complex integer ratios. The saliency of the downbeat is usually elicited by variations of loudness, pitch, or timbre in the perceived sound. It can also be generated endogenously via an active top-down process of rhythmic perception (Nozaradan et al., 2011; London, 2012). This endogenous sense of regular alternations of strong and weak patterns is commonly termed as *subjective metricization* (Keller, 2012). In short, meter induction organizes periodic beats in a hierarchical manner and can be modulated voluntarily.

Recent research has advanced in the identification of neural correlates of beat perception and meter induction. It has been demonstrated that neural activity increases at the frequencies corresponding to the beat and those corresponding to an induced meter (see Nozaradan et al., 2011, 2012). A frequency-tagging approach by means of electroencephalography (EEG) has been used to explore these neural substrates (Nozaradan, 2014; Nozaradan et al., 2015). This method uses periodic properties of the stimuli to induce steady-state evoked potentials (SS-EPs): changes in voltage that are stable in phase and amplitude over time. These neural responses can be elicited via different modalities (Vialatte and Maurice, 2009; Vialatte et al., 2010a; Nozaradan et al., 2012) and are easily recorded with EEG. Once SS-EPs are analyzed in the frequency domain, narrow-band peaks appear at the frequencies corresponding to the external stimuli. In addition to the emergence of these peaks reflecting bottom–up processing, those frequencies representing the beat and the meter are selectively enhanced. Crucially, top–down effects of meter induction are also captured, such as when meter is internally driven and imposed on the stimuli. When binary (march-like) and ternary (waltz-like) meter was mentally projected onto an auditory stimulus, Nozaradan et al. (2011) found an enhancement at the frequencies corresponding to the subharmonics of the beat: *f*/2 and *f*/3, respectively. Thus, the increase of neural activity at selected frequencies reflects the voluntarily control of meter induction, that is, the top–down processing that guides attention toward relevant points in the stimulus.

So far, the neural correlates of meter induction have primarily been studied in the auditory modality. There is an open debate about whether meter is restricted to the auditory modality or whether other modalities can access meter. Recent behavioral research has revealed that metrical structure is also apparent in visual stimuli. Lee et al. (2015) presented participants with videos of choreographies marking strong and weak beats at the same time as isochronous sounds. In their first experiment, deviant timbre sounds were presented in different metrical positions. Participants were slower in reacting to deviant sounds placed at the visually inferred strong positions, which suggests visual meter induction and a split of attention between modalities. Although beat and meter have been tested in other modalities, such as visual and tactile, these studies mostly involve motor acts, such as synchronized tapping (reviewed in Repp, 2005; Repp and Su, 2013). For example, it has been found that the act of moving at certain meter leads to listening preferences in infants (Phillips-Silver and Trainor, 2005, 2008). Movement also shapes the internal representation of auditory rhythms by enhancing the EEG signal at the metrical frequency (Chemin et al., 2014). However, it is difficult to disentangle the relative contribution of the motor act itself from metrical effects in these sensorimotor synchronization (SMS) studies.

There are good reasons to believe that beat processing and meter induction may be amodal. Several species have been found to effectively use interval-based and beat-based mechanisms in both the auditory and the visual modalities (Repp, 2005; Repp and Su, 2013; Patel and Iversen, 2014; Ravignani et al., 2014; Merchant et al., 2015). For example, visual rhythm synchronization has been attested in the behavior of some insects, such as fireflies (Buck and Buck, 1968), and in controlled laboratory studies with non-human primates, such as macaques (Zarco et al., 2009). Moreover, the accurate synchronizations observed during dance and music across human cultures (Fitch, 2013) suggests that these timing mechanisms might not only engage the visual modality, but also make use of a non-domain-specific meter.

The possibility that rhythm synchronization engages a domain-general timing mechanism makes it necessary to explore the extent to which we can also identify neural correlates for meter induction across modalities. The present work aims to explore meter induction in the visual modality by comparing the effects of endogenously driven meter projected onto auditory and visual periodic stimuli. We apply a frequency-tagging approach to the SS-EPs elicited during meter induction without the involvement of motor behavior. Our objective is to explore the extent to which meter induction is domain-specific: whether it is tightly constrained to the auditory modality, or whether similar neural correlates can be found across modalities. As visually transmitted meter is observed in some natural settings, such as musicians following a conductor in an orchestra, or dancers synchronizing with each other, our prediction is that we should observe similar metrical effects in both the auditory and the visual modalities.

## Materials and Methods

### Participants

Sixteen healthy musicians were included in the present study (9 females, 6 left-handers, mean age: 23.38 ± 3.85, age range: 18–35). There were 17 participants in total, but one male was excluded due to an excess of artifacts in the EEG data. All participants had extensive musical experience, starting at 6.31 ± 2.30 years of age, and six of them reported some training in dance. We chose to recruit musicians because it is clear to them what differentiates binary from ternary meter and because they have extensive experience extracting metrical cues from audiovisual sequences of rhythms. No participant reported any history of hearing, visual, motor, or psychiatric disorders, and all participants had normal or corrected-to-normal vision. All participants signed a written consent form and received payment for their participation in the study.

### Ethics Statement

All procedures were approved by the ethical committee from the Universitat Pompeu Fabra.

### Stimuli

**FIGURE 1:**
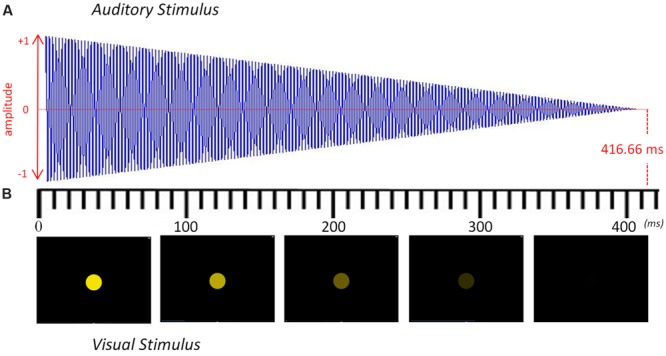
**Representation of the auditory and visual stimuli over time. (A)** Sound envelope of the auditory stimulus modulated from maximum to minimum volume. **(B)** Five frames of the visual stimulus depicting the modulation from maximum to minimum luminescence. Each stimulus lasted 416.66 ms and was repeated 84 times to yield a 35-s sequence with a steady beat at 2.4 Hz. The pitch or the color was changed after every 35-s sequence to maintain participants’ attention.

Stimuli consisted of isochronous sequences of either tones (in the auditory condition) or flashes (in the visual condition). They were presented at a frequency of 2.4 Hz (IOI = 416.66 ms). Every sequence lasted for 35 s and was comprised of 84 tones or flashes. All the target frequencies fell within the ecological range of tempo perception and production (Vialatte and Maurice, 2009; Nozaradan et al., 2011). Every event (tone or flash) within the sequence progressively diminished until the next one appeared (see **Figure 1**), thus marking the onset of the beat with the maximum intensity of sound (in the auditory condition) or with the maximum intensity of light (in the visual condition). Each condition had eight auditory or visual 35-s sequences. After each 35-s sequence, the pitch or the color of the stimuli was changed in order to maintain participants’ attention. Within each 35-s sequence, the stimuli were the same.

The auditory stimuli were presented at a comfortable hearing level through two speakers placed 70 cm in front of the participant. We converted a pure sinusoidal tone into stereo by using Audacity. These pure tones were raised half a tone in each 35-s sequence, going from an F4 up to a C5. The entire auditory condition, therefore, consisted of eight different sequences of 84 repeated sinusoidal tones: F4 (349.2 Hz), F#4 (370.0 Hz), G4 (392.0 Hz), G#4 (415.3 Hz), A4 (440.0 Hz), A#4 (466.2 Hz), B4 (493.9 Hz) and C5 (523.3 Hz).

The visual stimuli were presented on a computer screen (1280 × 1024 resolution) placed 70 cm in front of the participant. A colored circle was placed at the center of the screen with a black background and had a radius of 25 mm. To create the blinking effect, we progressively diminished its luminescence until it turned completely black (see **Figure 1**). These flashes changed color after every 35-s sequence of 84 flashes with the following RGB progression: red (255 0 0), orange (255 128 0), yellow (255 255 0), green (128 255 0), turquoise (0 255 128), light blue (0 255 255), dark blue (0 0 255), and violet (128 0 255). The auditory and visual stimuli were created using Matlab (v.2013, The MathWorks) and presented with Psychophysics Toolbox extensions (Brainard, 1997; Pelli, 1997).

### Procedure

Participants were seated in a comfortable armchair in a soundproof room with the keyboard placed on their lap. Our study consisted of three conditions: the passive beat condition (which served as a control), the binary imagery task, and the ternary imagery task. These three conditions were the same for the auditory and visual modalities. They were pseudo-randomly presented to each participant, with the control condition always presented first, and the visual and auditory modalities alternated, forming a total of 16 possible combinations. During the control condition, the participants were passively either looking at the flashes or listening to the sounds. In order to avoid the induction of an involuntary meter in the control condition, the well-known *tick-tock* effect (Brochard et al., 2003), we reminded the participants at the beginning of each 35-s sequence to perceive each beat individually, that is, as independent from the previous and the following tone or flash. During the binary and ternary tasks, in contrast to the control condition, participants were asked to mentally project a binary structure (strong–weak pattern) or a ternary structure (strong–weak–weak pattern) onto the same perceptual stimuli presented during the control condition. In other words, they were asked to silently project a metrical structure focusing on the subharmonics of the beat: *f*/2 (1.2 Hz) for the binary meter and *f*/3 (0.8 Hz) for the ternary meter. Participants were asked to start the meter imagery task as soon as the first stimulus was presented. At the end of each 35-s sequence, they had to report whether the last beat was strong or weak as a way of maintaining their attention and making sure they were focused on the task. Because the participants had to press the space bar to go on to the next block, they were allowed to take a break when needed. Once the study finished, a questionnaire was given to the participant to evaluate and comment on the stimuli and the difficulty of each task. Psychtoolbox was used to run the experiment.

### Electrophysiological Recording

The EEG signal was recorded using a BrainAmp amplifier and the BrainVision Analyzer Software package (v.2.0; Brain Products) using an actiCAP with 60 electrodes placed on the scalp according to the International 10/10 system (Fp1, Fp2, AF7, AF3, AF4, AF8, F7, F3, F1, Fz, F2, F4, F8, FT9, FT7, FT8, FT10, FC5, FC3, FC1, FC2, FC4, FC6, C5, C3, C1, Cz, C2, C4, C6, T7, T8, TP9, TP7, TP8, TP10, CP5, CP3, CP1, CPz, CP2, CP4, CP6, P7, P5, P3, P1, Pz, P2, P4, P6, P8, PO9, PO3, POz, PO4, PO10, O1, Oz, O2). Vertical and horizontal eye movements were monitored using two electrodes placed on the infra-orbital ridge and the outer canthus of the right eye. Two additional electrodes were placed on the left and right mastoid. The signals were referenced to the FCz online channel and all electrode impedances were kept below 25 kΩ. The signals were amplified and digitized at a sampling rate of 1000 Hz.

### Data Analyses

Preprocessing of the continuous EEG recordings was implemented using BrainVision Analyzer 2.1 (Brain Products GmbH). First, any channel that appeared flat or noisy was interpolated from the surrounding channels via spherical spline interpolation. All the channels were then filtered using a zero-phase Butterworth filter to remove slow drifts in the recordings, with a high pass filter at 0.1 Hz (48 dB/oct) and low pass filter at 10 Hz (time constant 1.591549, 48 dB/oct). Channels with EEG exceeding either ±100 μV at any channel, activity <0.5 μV, or voltage step/sampling >50 μV within intervals of 200 ms, were automatically detected oﬄine. Subsequently, eye blinks and muscular movements were corrected using Ocular Correction ICA. Finally, the filtered EEG data was segmented into epochs of 36 s (corresponding to the 35-s sequences with an extra second in the beginning) for each condition and modality. These files were then exported to Matlab. All further analyses were performed in Matlab and SPSS (version 19, IBM).

For each condition and modality, eight epochs lasting 32.5 s were obtained by removing the first 3.5 s of each sequence. This removal, as justified in Nozaradan et al. (2011), discards the evoked potential related to the stimuli onset and relies on the fact that SS-EPs require several repetitions or cycles to be elicited (Repp, 2005; Vialatte et al., 2010a). In order to enhance the signal-to-noise ratio and attenuate activities that are not phase locked to the auditory and visual stimuli, the EEG epochs for each participant, modality, and condition were averaged across trials. To get the signal’s amplitude (in μV), we applied a fast Fourier transform (FFT), and to get its power (in μV^2^), we squared the modulus of the FFT. Both frequency spectra ranged from 0 to 500 Hz with a frequency resolution of 0.0305 Hz. The obtained signal is assumed to correspond to the EEG activity induced by the physical stimuli and the meter imagery. However, it may also include residual background noise due to spontaneous activity. Two different signal-to-noise techniques were applied: the subtraction method used in Nozaradan et al. (2011) for the amplitude spectrum (in μV) and a relative measure that compared each individual’s binary and ternary meter values to their own beat condition as a baseline for the power spectrum (converting μV^2^ into decibels), thus minimizing variation due to group variability.

For the amplitude spectrum, noise was removed by subtracting the averaged amplitude of the two surrounding non-adjacent frequency bins, ranging from -0.15 to -0.09 Hz and from 0.09 to 0.15 Hz, at each frequency bin from 0.5 to 5 Hz. For the power spectrum, we assume that the beat condition serves as a baseline, since the subjects were passively listening to the stimuli, and that their EEG activity corresponds to the processing of beat without any top–down projection of meter. We took the power spectrum of each meter imagery condition for each participant and divided it by their own control condition. Subsequently, we converted these values into dB by taking the log_10_ of each value and multiplying by 10. Following this procedure, we do not need to compare the selected frequency bins among conditions and modalities against the control, but instead check whether the obtained values at each frequency of interest differ from zero. This procedure makes the comparison between modalities more reliable, as the effect of meter is relative to the control condition of each modality.

In order to correct for spectral leakage from our target frequencies in the amplitude spectrum, we averaged every target frequency bin ±0.0305 Hz, the three frequency bins centered on the target frequencies, as Nozaradan et al. (2011) did. However, to compare between the peaks, we only took the values from every target frequency bin. We did not apply this technique for spectral leakage to the original power spectrum because, after using the baseline correction method, the induced activity was centered very concisely in a single bin. The mean of all electrodes across each individual’s scalp was calculated for each condition and modality at each target frequency, revealing multiple peaks in the data. For the amplitude spectra, the values for each target frequency (0.8, 1.2, 2.4, 4.8 Hz) were separately submitted to two-way repeated measures ANOVAs with the factors Modality (auditory and visual) and Condition (beat, binary, ternary). When one of the ANOVA factors was significant, *post hoc* pairwise comparisons were performed with Fischer’s LSD and Bonferroni. Size effects were expressed using the partial η^2^. For the power spectra, one sample *t*-tests were used to see if the values at each target frequency significantly differed from zero. The significance level was set at *p* < 0.05 for all statistical analyses.

The present approach does not deal with topological effects because our hypothesis does not predict any region of interest for a domain-general meter induction, although certain correlations could appear (such as occipital areas showing a strong connection to the visual modality of the stimuli). This is the reason why the means from every electrode were averaged across the scalp, thereby excluding selection biases.

## Results

### Amplitude Spectra

**FIGURE 2:**
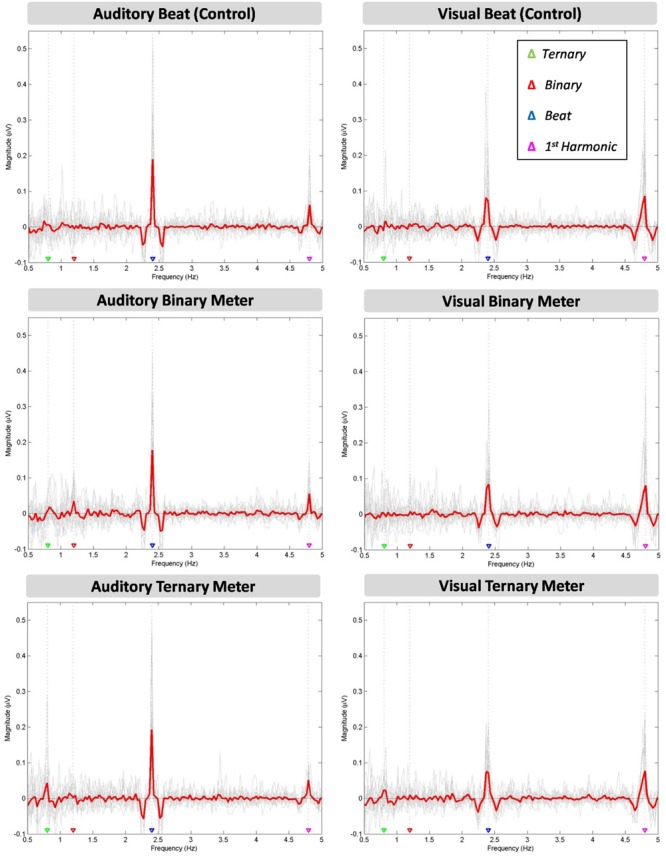
**Six amplitude spectra depicting the amplitude (μV) of the averaged EEG signal at each frequency between 0.5 and 5 Hz.** The auditory **(left column)** and visual **(right column)** modalities are split into three conditions: the beat control (first row), the binary meter imagery task (second row), and the ternary meter imagery task (third row). The mean of all participants’ amplitudes (gray lines) is depicted in red for each condition and modality. The frequencies of the ternary meter, binary meter, beat, and its first harmonic are signaled with gray dotted lines and colored triangles over the abscissa: 0.8 Hz (green), 1.2 Hz (red), 2.4 Hz (blue) and 4.8 Hz (magenta).

In **Figure 2**, the mean of all participants’ amplitudes (red line) is plotted over each individual’s amplitude spectra (gray lines) at the target frequencies (0.8, 1.2, 2.4, 4.8 Hz). A clear peak appears at the frequency of the stimuli (2.4 Hz) in all the conditions (beat, binary, ternary). Similarly, a peak is observed for the first harmonic (4.8 Hz) in all three conditions. However, there seem to be differences across conditions regarding the sub-harmonics. The peak at 1.2 Hz only appears in the auditory binary condition, while the peak at 0.8 Hz is found in both auditory and visual ternary conditions. Furthermore, there is a larger peak at the beat frequency for all three auditory conditions compared to their visual analogs, whereas the inverse effect occurs at the frequency of the first harmonic, depicting a larger peak for all three visual conditions.

A two-way repeated measures ANOVA [Modality (auditory and visual) × Condition (beat, binary, ternary)] was applied to each frequency of interest (0.8, 1.2, 2.4, 4.8 Hz) separately. **Table 1** reports the values obtained from ANOVAS for Modality, Condition, and their interaction. For the frequency of the first harmonic of the beat (4.8 Hz), there was a main effect of Modality: *F*_(1,15)_ = 14.945, η^2^ = 0.499, *p* = 0.002. For the ternary subharmonic of the beat (*f*/3 = 0.8 Hz), a main effect of Condition was observed [*F*_(2,30)_ = 5.018, η^2^ = 0.251, *p* = 0.013]. However, for the binary subharmonic of the beat (*f*/2 = 1.2 Hz), no main effect of Condition was found [*F*_(2,30)_ = 1.599, η^2^ = 0.096, *p* = 0.219]. *Post hoc* pairwise comparisons (see **Table 2**) using a Bonferroni adjusted alpha indicated that (i) the averaged amplitudes of the ternary condition were greater than those of the beat control at 0.8 Hz (MD = 0.454, *p* = 0.046), and (ii) the averaged amplitudes of the visual modality were greater than those of the auditory modality at 4.8 Hz (MD = 1.091, *p* = 0.002). Interestingly, less restrictive *post hoc* pairwise comparisons using Fischer’s Least Significant Difference (LSD) showed that the averaged amplitudes of the ternary condition were also greater than those of the binary condition at 0.8 Hz (MD = 0.434, *p* = 0.022).

**Table 1:** Results of the four two-way repeated measures ANOVAs applied to the averaged amplitudes (3 bins) of each frequency of interest: 0.8, 1.2, 2.4, and 4.8 Hz.

	*df*	*F*	*p*	η^2^
**0.8 Hz**				
Modality	1, 15	1.890	0.189	0.112
Condition	2, 30	5.018	0.013^∗^	0.251
Modality ^∗^ Condition	2, 30	0.206	0.815	0.014
**1.2 Hz**				
Modality	1, 15	2.468	0.137	0.141
Condition	2, 30	1.599	0.219	0.096
Modality ^∗^ Condition	2, 30	1.876	0.191	0.111
**2.4 Hz**				
Modality	1, 15	1.662	0.217	0.100
Condition	2, 30	0.214	0.809	0.014
Modality ^∗^ Condition^1^	1.343, 20.138	0.497	0.542	0.032
**4.8 Hz**				
Modality	1, 15	14.945	0.002^∗∗^	0.499
Condition	2, 30	0.564	0.575	0.036
Modality ^∗^ Condition	2, 30	0.309	0.736	0.020

*The two levels of Modality (auditory and visual) and Condition (beat control, binary-meter, and ternary-meter) are shown accompanied by their interactions. For each level and interaction, degrees of freedom, *F*-statistics, *p*-values, and size effects are reported. ^1^A Greenhouse–Geisser correction was applied to correct for violations of sphericity. ^∗^*p* < 0.05, ^∗∗^*p* < 0.01.*

**Table 2:** *Post hoc* pairwise comparisons from the two-way repeated measures ANOVAs of the averaged amplitudes (3 bins) with and without adjustments for multiple comparisons.

			Mean difference	Standard error	Significance	
					Fischer’s LSD	Bonferroni
**0.8 Hz**	**Condition**	**Condition**				
	Bin.	Beat	0.020	0.150	0.897	1
	Tern.	Beat	0.454	0.166	0.015^∗^	0.046^∗^
	Tern.	Bin.	0.434	0.169	0.022^∗^	0.065
**4.8 Hz**	**Modality**	**Modality**				
	Aud.	Vis.	-1.091	0.282	0.002^∗∗^	0.002^∗∗^

*For the frequencies 0.8, 2.4, and 4.8 Hz, the mean differences, standard deviations, and *p*-values (Fischer’s LSD as non-readjusted alpha, Bonferroni as readjusted alpha) are reported. Significance level was always kept below 0.05. The condition ‘Beat’ stands for the beat control, the condition ‘Bin.’ stands for the binary meter, and the condition ‘Tern.’ stands for the ternary meter. The modality ‘Aud.’ stands for audition, and the modality ‘Vis.’ stands for vision. ^∗^p < 0.05, ^∗∗^p < 0.01.*

We found modality differences regarding the first harmonic of the beat (4.8 Hz), but not regarding the beat (2.4 Hz), even though the peaks were apparently higher in the auditory modality (see **Figure 2**). This counterintuitive finding may be related to the way we averaged the amplitudes for each peak, which assumed that a considerable leakage was taking place. However, the amplitude spectra show that the peak for the beat in the auditory modality was sharper and larger, while in the visual modality was wider and shorter.

A second two-way repeated measures ANOVA [Modality (auditory and visual) × Condition (beat, binary, ternary)] was applied to the top-of-the-peak values of each frequency of interest (0.8, 1.2, 2.4, 4.8 Hz) separately, as reported in **Table 3**. For the frequency of the beat (2.4 Hz), there was a main effect of Modality: *F*_(1,15)_ = 14.215, η^2^ = 0.487, *p* = 0.002. For the ternary subharmonic of the beat (*f*/3 = 0.8 Hz), a main effect of Condition was observed [*F*_(1.345,20.178)_ = 5.842, η^2^ = 0.280, *p* = 0.018]. For the binary subharmonic of the beat (*f*/2 = 1.2 Hz), a main effect of Condition [*F*_(1.422,21.334)_ = 4.609, η^2^ = 0.235, *p* = 0.032] and an interaction were attested [*F*_(2,30)_ = 1.783, η^2^ = 0.106, *p* = 0.026]. *Post hoc* pairwise comparisons (see **Table 4**) using a Bonferroni adjusted alpha indicated that (i) the peak amplitudes of the ternary condition were higher than those of the beat control at 0.8 Hz (MD = 1.178, *p* = 0.018), (ii) the peak amplitudes of the binary condition were higher than those of the beat control at 1.2 Hz (MD = 0.655, *p* = 0.050), and (iii) the peak amplitudes of the auditory modality were higher than those of the visual modality at 2.4 Hz (MD = 3.592, *p* = 0.002). Less restrictive *post hoc* pairwise comparisons using Fischer’s LSD showed that the peak amplitudes of the binary condition were significantly higher than those of the beat control at 1.2 Hz (MD = 0.655, *p* = 0.017).

**Table 3:** Results of the four two-way repeated measures ANOVAs applied to the top-of-the-peak amplitudes (1 bin) at 0.8, 1.2, 2.4, and 4.8 Hz.

	*df*	*F*	*p*	η^2^
**0.8 Hz**				
Modality	1, 15	3.269	0.091	0.179
Condition^1^	1.345, 20.178	5.842	0.018^∗^	0.280
Modality ^∗^ Condition^1^	1.445, 21.669	0.309	0.667	0.020
**1.2 Hz**				
Modality	1, 15	0.161	0.694	0.011
Condition^1^	1.422, 21.334	4.609	0.032^∗^	0.235
Modality ^∗^ Condition	2, 30	4.134	0.026^∗^	0.216
**2.4 Hz**				
Modality	1, 15	14.215	0.002^∗∗^	0.487
Condition	2, 30	0.042	0.959	0.003
Modality ^∗^ Condition	2, 30	1.783	0.202	0.106
**4.8 Hz**				
Modality	1, 15	0.748	0.401	0.047
Condition	2, 30	0.902	0.416	0.057
Modality ^∗^ Condition	2, 30	0.007	0.993	0.000

*The two levels of Modality (auditory and visual) and Condition (beat control, binary-meter, and ternary-meter) are shown accompanied by their interactions. For each level and interaction, degrees of freedom, *F*-statistics, *p*-values, and size effects are reported. ^1^A Greenhouse–Geisser correction was applied to correct for violations of sphericity. ^∗^p < 0.05, ^∗∗^p < 0.01.*

**Table 4:** *Post hoc* pairwise comparisons from the two-way repeated measures ANOVAs of the top-of-the-peak amplitudes (1 bin) with and without adjustments for multiple comparisons.

			Mean difference	Standard error	Significance	
					Fischer’s LSD	Bonferroni
**0.8 Hz**	**Condition**	**Condition**				
	Bin.	Beat	0.250	0.225	0.284	0.852
	Tern.	Beat	1.178	0.369	0.006^∗∗^	0.018^∗^
	Tern.	Bin.	0.929	0.458	0.061	0.182
**1.2 Hz**						
	Bin.	Beat	0.655	0.243	0.017^∗^	0.050^∗^
	Tern.	Beat	0.145	0.141	0.320	0.961
	Tern.	Bin.	-0.510	0.274	0.083	0.248
**2.4 Hz**	**Modality**	**Modality**				
	Aud.	Vis.	3.592	0.953	0.002^∗∗^	0.002^∗∗^

*For the frequencies 0.8, 2.4, and 4.8 Hz, the mean differences, standard deviations, and *p*-values (Fischer’s LSD as non-readjusted alpha, Bonferroni as readjusted alpha) are reported. Significance level was always kept below 0.05. The condition ‘Beat’ stands for the beat control, the condition ‘Bin.’ stands for the binary meter, and the condition ‘Tern.’ stands for the ternary meter. The modality ‘Aud.’ stands for audition and the modality ‘Vis.’ stands for vision. ^∗^p < 0.05, ^∗∗^p < 0.01.*

These results might be due to a similar top–down meter effect for both modalities in the ternary-meter condition enhancing the ternary subharmonic of the beat (*f*/3), the downbeat of the ternary meter. Unfortunately, this enhancement was not consistently found for the binary subharmonic (*f*/2) because the averaged values of both auditory and visual binary-meter conditions at 1.2 Hz might not be significantly greater than those of other conditions. In fact, the interaction reported by the second ANOVA (comparing each top-of-the-peak value) at 1.2 Hz points to the absence of the binary-meter effect in one modality. Furthermore, both binary and ternary metrical effects could also be affected by the variability across participants in projecting meter or in unconsciously grouping the beat during the control condition, despite our instructions. In order to control for inter-individual variability (see the gray lines of **Figure 2**), we explored our data using a different signal-to-noise method, namely using each individual’s beat condition as a baseline in order to obtain a relative measure that takes that person’s variability into account.

### Power Spectra

**FIGURE 3:**
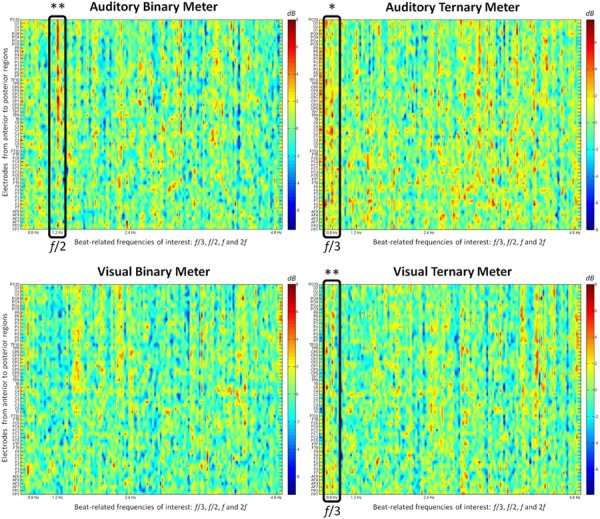
**The relative value (from -7 to 8 dB) obtained after subtracting the power spectrum (μV^2^/Hz) of the control condition from each metrical condition is shown at each electrode.** The frequencies of interest (0.8, 1.2, 2.4, and 4.8 Hz) appear on the frequency axis (from 0.6 to 5 Hz). First row: auditory conditions. Second row: visual conditions. First column: binary meter task. Second column: ternary meter task. Black rectangles frame the relevant frequencies when their values are significantly distinct from zero. The electrodes are ordered from anterior to posterior regions, with odd and even numbers representing the right and left hemispheres, respectively. ^∗^ *p* < 0.05, ^∗∗^ *p* < 0.01.

The power spectra for each modality and condition were obtained by taking the modulus squared of the amplitudes resulting from the FFT. Here, we used the control (beat) condition as a baseline to normalize and convert the amplitudes from the metrical conditions (binary, ternary) into decibels. This procedure consisted of applying the following operation: 10 log_10_ 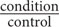. This method gives us the opportunity to see relative distances from zero as differences between conditions with respect to the control. When positive, the values indicate more power for the metrical condition, whereas when negative, they indicate more power for the beat baseline. **Figure 3** shows all the relativized power spectra displaying the activity of all the electrodes at each frequency.

**FIGURE 4:**
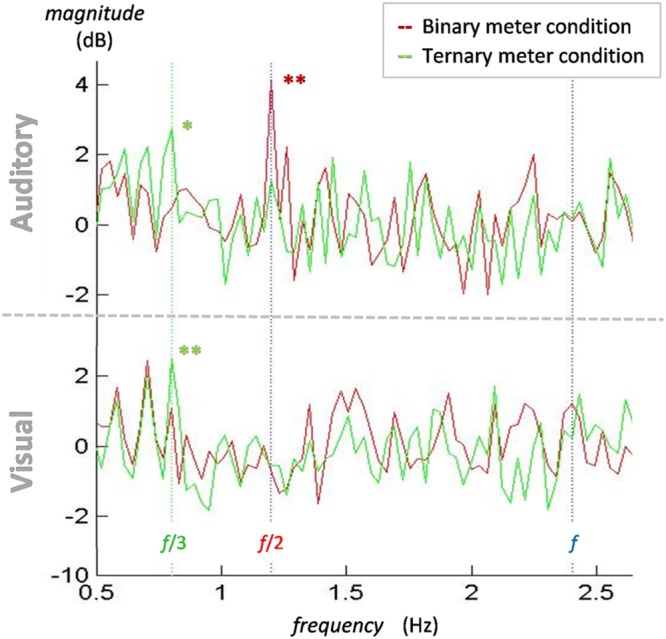
**Normalized power spectra of the binary and ternary conditions for the mean of all participants and electrodes in both the auditory and the visual modalities.** In both auditory **(top)** and visual **(bottom)** modalities, a peak for the ternary condition (green line) appears at 0.8 Hz (green dotted line). A peak for the binary condition (red line) at 1.2 Hz (red dotted line) is only observed in the auditory modality. There is no contrasting peak at 2.4 Hz (blue dotted line) in either modality. ^∗^*p* < 0.05, ^∗∗^*p* < 0.01.

To summarize the differences between conditions and modalities, **Figure 4** depicts the mean of all participants’ electrodes contrasting each condition in both the visual and the auditory modality. The frequencies of interest are marked with a vertical line to make it easier to see the meter-induced peaks at the subharmonics of the beat. Similar to the findings in the amplitude spectra, there are larger peaks at 0.8 Hz for the ternary condition in both modalities, but there is a peak at 1.2 Hz for the binary condition in the auditory modality only. In contrast with our first analyses, no differences arise between modalities at the frequency of the beat (2.4 Hz) in the power spectra. This is due to the use of the beat condition as a baseline. Given that beat is similarly induced in all conditions, dividing the two conditions will result in a relative value close to zero.

One-sample *t*-tests were applied to the spectrum of each target frequency to examine whether the values significantly differed from zero. At 0.8 Hz, the values of the ternary conditions were significantly different from zero in the auditory [*t*_(15)_ = 2.778, *p* = 0.014] and the visual [*t*_(15)_ = 3.692, *p* = 0.002] modalities. The same occurred at 1.2 Hz for the values of the binary condition in the auditory modality [*t*_(15)_ = 3.489, *p* = 0.003]. These findings show that the peaks appearing in both ternary conditions at 0.8 Hz and the auditory binary condition at 1.2 Hz are significantly different from zero and thus larger than the control condition. However, no effects for the visual binary meter were attested.

## Discussion

Our work is among the first studies to compare meter induction between two different modalities, audition and vision, without requiring synchronized, overt movement. Although SMS studies have also explored the degree to which participants can reliably extract the pulse from visual rhythms (Patel et al., 2005; Repp, 2005; Nozaradan et al., 2012; Repp and Su, 2013; Iversen et al., 2015), there is not much information to date regarding meter induction in other modalities which do not involve motor acts. Here, we examined whether visually induced beats can be organized in endogenously driven hierarchies. To this end, each participant was asked to internally project binary and ternary meter onto the isochronous stimuli. Their recorded EEG data was converted into the frequency domain, resulting in amplitude and power spectra. We observed an effect of ternary meter induction in both the auditory and the visual modalities, as well as an effect of binary meter induction in the auditory modality. This suggests that some degree of meter induction can also be observed in the visual modality.

We evaluated the SS-EPs elicited by the beat frequency and its natural first harmonic in both modalities. The emergence of a periodic entrainment at the frequency of the beat’s first harmonic (2*f =* 4.8 Hz) illustrates a natural preference for integer harmonics that tend to appear involuntarily when a periodic stimulus is presented. In fact, this entrainment may be related to a predisposition to subdivide the beat into integer harmonics, like duplets (1:2) or triplets (1:3) of eighth notes in music. We observed different amplitudes at the frequency of the beat and its first harmonic depending on the stimuli’s modality. There was a significantly higher peak at 2.4 Hz in the auditory modality than in the visual modality. Inversely, there was a significantly greater peak at 4.8 Hz in the visual modality than in the auditory modality. Since our visual stimuli did not abruptly appear and disappear at 4.8 Hz, but progressively vanished (see **Figure 1**), there is no reason to believe that the stimulus’ offset reinforced the first harmonic in the visual domain. This difference across modalities could be related to the comfortable frequencies that have been found for vision and audition with synchronized tapping (Repp, 2003). While the upper inter-onset interval (IOI) would be the same for both modalities (around 1800 ms), the lower IOI seems to be just above 460 ms for visual flashes and 160 ms for auditory beats (Repp, 2005). These modality-specific boundaries for SMS could reflect the neural demands of extracting and keeping the beat in each modality, possibly working on different frequencies. Importantly, the difference across modalities could also be due to the fact that musicians are well-trained to deal with auditory beats, and may be able to accurately synchronize with the frequency of the auditory stimuli, as shown by the sharper peak at the auditory beat (**Figure 2**). In contrast, since musicians may be less used to visual beats, their synchronization with the frequency of the visual stimuli may become less accurate and more distributed around 2.4 Hz, as shown by the wider, rounder peak (**Figure 2**). In this case, experience may play an important role in determining the differences between modalities.

A key finding in the present study is the emergence of periodic responses at the subharmonics *f*/2 and *f*/3 of the beat. These effects reflect the voluntary metrical interpretation of the beat in the binary and the ternary conditions. Our results suggest that neural entrainment in both modalities not only occurs for the beat, but also for the subharmonic *f*/3 during the ternary condition. This finding supports top–down effects of meter induction in the visual modality. However, we did not observe any effect of the binary meter in the visual modality. This lack of effect in the binary condition suggests that there is no automatic conversion between the visual and the auditory modalities. If there were such a conversion, we should observe an effect of binary and ternary meter on both modalities (see Guttman et al., 2005; McAuley and Henry, 2010). Nevertheless, the fact that meter induction is apparent in the visual modality for the ternary, but not for the binary, meter condition suggests that meter induction applies to the visual cues independent of any auditory conversion. To confirm this idea, future research testing visual meter induction in deaf people is needed, as a visual-to-auditory conversion would not be expected in this case. Furthermore, some participants reported in the questionnaire that, instead of “counting” the pulse or thinking metrical patterns in the visual conditions, they made use of visual cues to project the meter onto the flashes, such as imagining the downbeat brighter or slightly displaced toward one side of the screen. The alteration of these visual features to project the metrical structure may also provide evidence against an auditory-to-visual conversion.

The fact that we did not find evidence of meter induction in the visual binary condition could be due to different cognitive demands across the subharmonics of the beat. A magnetoencephalographic study conducted by Fujioka et al. (2010) revealed differences in internally inducing binary and ternary meter in the auditory domain. Distinct time courses of auditory evoked responses on distributed networks were found for binary and ternary meter. The authors also observed that the contrast between strong and weak beats was only present for the ternary meter. Thus, differences between projecting binary and ternary meter could have interacted with the modality we used to induce the beat. Although more research is needed to clarify the differences between binary and ternary meter in terms of cognitive demands, the results we observed from the visual ternary condition support the idea that meter induction can apply beyond the auditory modality.

There is a feature in our visual stimuli that could have contributed to the lack of an effect in the binary condition. We used colored flickering flashes that suddenly appeared and gradually vanished to promote a beat. Stimuli such as flashes have been used extensively to create SS-EPs (Krause et al., 2010; Vialatte et al., 2010b) and even seem to be perceived with binary meter by deaf individuals (Iversen et al., 2015). However, recent studies suggest it is easier to observe SMS in the visual domain using geometrical and spatial features (Hove et al., 2010), as well as stimuli displaying natural and biological motion (Hove et al., 2013b; Su, 2014a,b). Showing a bouncing ball on a screen, Gan et al. (2015) obtained slightly better results on visual synchronization at IOIs from 500 to 900 ms compared to using an auditory metronome. Similarly, Iversen et al. (2015) reported equally accurate performance by deaf and hearing individuals when they had to synchronize with a ball bouncing at an IOI of 600 ms. In light of these findings, we can speculate that using motion cues could have aided the neural synchronization with the beat and perhaps facilitated the projection of the binary meter in the visual modality, whose frequency actually fell within the above-mentioned IOI ranges.

It has been proposed that meter consists of a cyclical fluctuation of attention over isochronous events to yield expectancies and predictions of incoming beats (Jones and Boltz, 1989; Large and Jones, 1999). A mirroring oscillatory network could explain the neuronal engagement to the external stimuli, either auditory or visual, such as the non-linear oscillator network proposed by the Resonance Theory (Large, 2008, 2010; Large et al., 2015). In the visual domain, these predictions would be fundamental to allow for dance synchrony and other coordinated social activities, such as sports. Studies using neuroimaging techniques provide further support for cross-modal meter. For instance, Hove et al. (2013a) found that the basal ganglia activity was more associated with SMS stability than with modality features. Musical meter has also been found to elicit cross-modal attention effects in the caudate nucleus (Trost et al., 2014). Coupled with our findings on the ternary visual meter, the general picture appears to be that of an amodal timing mechanism for beat and meter, allowing humans to deal with temporal information across modalities.

Comparative cognition studies also point toward the idea that timing mechanisms are more detached from a single modality than previously believed. Trained rhesus monkeys (macaques) showed an accurate performance of a synchronization-continuation task with both visual and auditory metronome at different tempos (Zarco et al., 2009), but their tapping behavior was clearly biased toward visual cues (Merchant and Honing, 2013). In addition, Japanese macaques were found to synchronize limb movements when facing each other, suggesting social coordination through visual imitation (Nagasaka et al., 2013). In our closest relatives, the chimpanzees, synchronization has been tested in the auditory domain (Hattori et al., 2013). However, likely by using both visual and auditory information, a bonobo intermittently displayed entrainment and phase matching to distinct isochronous sounds when interactively drumming with a human drummer (Large and Gray, 2015). Rhythmic behaviors, such as the drumming found in wild gorillas and chimps, seem to be tied to social functions (Ravignani et al., 2013). These findings suggest that primates may have evolved social interactive behaviors (i.e., the ancestors of our music and dance) by gradually tuning the neurodynamics of their timing mechanisms toward a more precise visuo- and audio-motor coupling so as to improve social learning, group coordination, and cohesion. Among primates, only humans are complex vocal learners, an ability that requires tighter auditory-motor connections (Petkov and Jarvis, 2012; Patel and Iversen, 2014; Merchant et al., 2015) than those found in the motor cortico-basal ganglia-thalamo-cortical circuit of primates (Merchant and Honing, 2013). We propose that the advantages of our evolved beat-based timing mechanism are not restricted to the auditory modality. Instead, even though the auditory modality has been specialized for the rhythms of speech and music through cultural experience, these same mechanisms may also be available to process rhythms in other modalities, allowing for sign language, dance, and sports.

The present results suggest that meter induction is domain-independent. A top-down projection of meter, without having any external cue to mark the metrical structure, is available for both the visual and the auditory modalities. This is informative regarding current theories on rhythm evolution and opens interesting questions. If meter induction evolved as a result of purely acoustic rhythm processing, it is then necessary to explain how it emerges in the visual modality. One possibility is that the feeling of meter emerges after linking the kinesthetic and vestibular systems to another perceptual modality, like vision or audition. In fact, the importance of the vestibular system was highlighted by the study of Phillips-Silver and Trainor (2005), which looked at 7-month-old babies’ metrical preferences after being bounced at binary and ternary meter. In addition, this vestibular and kinesthetic feeling of meter could also be the base for the movements found in musicians and dancers, and may point to how they learn to extract meter from distinct modalities. Another possibility, proposed by Fitch (2013), is that the hierarchical component of meter derives from a more general computation that is specialized in building temporal hierarchies. This would explain why meter is present in both music and language and why it could also be applicable to other modalities. If that were the case, the hierarchical organization of beats would be a by-product of our linguistic mind, and the organization of rhythm in music and dance may not be much different from the organization of rhythms in speech and signing, such as those constituting stress and prosody. Finally, it is still an open question whether animals can take advantage of this rhythmic mechanism that is not constrained to the auditory modality. The lack of evidence in the animal kingdom cannot be used as evidence that only humans have meter (Fitch, 2013), as several species show coordinated rhythmic abilities across modalities and deserve to be properly studied (Zarco et al., 2009; Nagasaka et al., 2013; Large and Gray, 2015).

Finally, it is important to consider the extent to which the present findings can be generalized to other populations. We tested musicians, who have been found to show an increased sensitivity to events and physical changes occurring in strong beat positions (Geiser et al., 2010; Repp, 2010; Kung et al., 2011; but see Bouwer et al., 2014). This suggests that musical expertise enhances attention to relevant metrical positions (Grahn and Rowe, 2009; Doelling and Poeppel, 2015). Accordingly, the extensive experience with auditory rhythmic stimuli in the population we tested could contribute to explain the variability in metrical timing observed in the visual domain. Future studies should test non-musicians in order to claim that meter induction is a cross-modal timing mechanism.

## Conclusion

The present study tackles the nature of musical meter, fundamental to the organization of events over time in a hierarchical way. We compared meter induction in audition and vision and found a similar effect for projecting ternary meter in both modalities. However, we only found an effect for binary meter in the auditory modality. The fact that ternary meter was successfully projected in both modalities suggests that human rhythmic abilities are more domain-general than previously believed. The existence of meter induction in the visual domain supports the idea of amodal timing mechanisms. These mechanisms seem to be at least partially present in some primates and may have developed in our species. It is the evolution of these amodal timing mechanisms that may have allowed us to master language, music, dance, and other synchronized activities that require a precise timing of actions across modalities.

## Author Contributions

AC-M designed and ran the study, analyzed the data, and wrote the article; RdM contributed to the design of the study, analyzing the data, and revising the article; JT designed the study and supervised the analysis of the data and the writing of the article.

## Conflict of Interest Statement

The authors declare that the research was conducted in the absence of any commercial or financial relationships that could be construed as a potential conflict of interest.
